# The embryonic expression pattern of a second, hitherto unrecognized, paralog of the pair-rule gene *sloppy-paired* in the beetle *Tribolium castaneum*

**DOI:** 10.1007/s00427-020-00660-x

**Published:** 2020-05-20

**Authors:** Ralf Janssen

**Affiliations:** Department of Earth Sciences, Palaeobiology, Uppsala University, Villavägen 16, 75236 Uppsala, Sweden

**Keywords:** Sloppy-paired, Slp, FoxG, Segmentation, Pair-rule, Segmental patterning, Forkhead

## Abstract

In the fly *Drosophila melanogaster*, a hierarchic segmentation gene cascade patterns the anterior-posterior body axis of the developing embryo. Within this cascade, the pair-rule genes (PRGs) transform the more uniform patterning of the higher-level genes into a metameric pattern that first represents double-segmental units, and then, in a second step, represents a true segmental pattern. Within the PRG network, primary PRGs regulate secondary PRGs that are directly involved in the regulation of the next lower level, the segment-polarity genes (SPGs). While the complement of primary PRGs is different in *Drosophila* and the beetle *Tribolium*, another arthropod model organism, both *paired* (*prd*) and *sloppy-paired* (*slp*), acts as secondary PRGs. In earlier studies, the interaction of PRGs and the role of the single slp ortholog in *Tribolium* have been investigated in some detail revealing conserved and diverged aspects of PRG function. In this study, I present the identification and the analysis of embryonic expression patterns of a second slp gene (called *slp2*) in *Tribolium*. While the previously identified gene, *slp*, is expressed in a typical PRG pattern, expression of *slp2* is more similar to that of the downstream-acting SPGs, and shows expression similarities to *slp2* in *Drosophila*. The previously reported differences between the function of *slp* in *Drosophila* and *Tribolium* may partially account for the function of the newly identified second slp paralog in *Tribolium*, and it may therefore be advised to conduct further studies on PRG function in the beetle.

**Electronic supplementary material:**

The online version of this article (10.1007/s00427-020-00660-x) contains supplementary material, which is available to authorized users.

## Introduction

In the dipteran fly *Drosophila melanogaster*, a hierarchic segmentation gene cascade almost synchronously controls the patterning of the early embryo along the anterior-posterior (AP) body axis into a defined number of single segments (reviewed in e.g. Akam 1987, Davis and Patel 2003). First, maternally provided factors form anterior to posterior and posterior to anterior morphogen gradients, that then regulate the zygotically expressed gap genes (GGs) (e.g. Fröhnhofer and Nüsslein-Volhard 1986). The GGs, which are expressed in broad overlapping domains along the AP axis of the developing embryo, regulate another group of zygotically expressed genes, the so-called pair-rule genes (PRGs). PRGs are typically expressed in the form of seven transversal stripes corresponding to alternating segment primordia (e.g. Jäckle et al. 1992). For most of the PRGs, these primary expression patterns change into a segmental pattern. Within the PRG network, the so-called primary PRGs such as *even-skipped* (*eve*) and hairy (*h*) act upstream of the so-called secondary PRGs, which in *Drosophila* are *sloppy-paired* (*slp*) and *paired* (*prd*) (reviewed in e.g. Clark 2017). In a complex combinatorial interaction, the PRGs then regulate the activity of the segment-polarity genes (SPGs), which act to maintain the parasegment borders, and define the polarity of each segment (e.g. Ingham et al. 1988, Cadigan et al. 1994a, recently reviewed in Clark et al. 2019).

This mode of segment formation, in which all segments are patterned synchronously at the blastoderm stage, is derived, and most other insects, as well as all other arthropods, only form their most anterior segments in a similar way (reviewed in Peel 2004). Posterior segments, however, are added from a posterior segment addition zone (SAZ). In most cases, single segmental units are added from the SAZ (e.g. Hughes and Kaufman 2002; Schoppmeier and Damen 2005; Janssen et al. 2011), but in some arthropods, all, or a number of segments, are added (or patterned) with a double-segment periodicity (e.g. Binner and Sander 1997; Dearden et al. 2002; Chipman et al. 2004; Erezyilmaz et al. 2009; Janssen et al. 2012; El-Sherif et al. 2012; Sarrazin et al. 2012). This latter mechanism is reminiscent of the initial expression of the PRGs in *Drosophila* and their pair-rule function. While it is still unclear if pair-rule patterning (and function) is a conserved ancestral trait of arthropod segmentation, it appears likely that the PRGs in general are involved in segmentation, as evident from functional studies (e.g. Liu and Kaufman 2005; Mito et al. 2007; Rosenberg et al. 2015; Xiang et al. 2017; Auman and Chipman 2018), and the analysis of gene expression patterns (e.g. Damen et al. 2000, 2005; Dearden et al. 2002; Hughes and Kaufman 2002; Chipman and Akam 2008; Janssen et al. 2011; Green and Akam 2013; Schönauer et al. 2016).

Compared with *Drosophila*, the beetle *Tribolium castaneum* displays a more conservative mode of development: The anterior segments are formed from the blastoderm, but posterior segments are added sequentially. In both the blastoderm that give rise to the anterior segments, and the posteriorly added segments, a clock-like mechanism including the function of PRGs appears to be involved. This mechanism generates/patterns segments with a double-segment periodicity (Choe et al. 2006; El-Sherif et al. 2012; Sarrazin et al. 2012), although the last-formed posterior segments indeed may be patterned one by one, at least on the level of SPGs (Janssen 2014). Generally, PRG orthologs have been in the focus of unravelling the segmentation mechanisms in *Tribolium* (e.g. Sommer and Tautz 1993, Brown et al. 1994, 1997, Maderspacher et al. 1998, Schröder et al. 2000, Eckert et al. 2004, Aranda et al. 2008, Bolognesi et al. 2009, Choe and Brown 2007, 2009, Peel et al. 2013, El-Sherif et al. 2014), and one of these genes of interest is the *sloppy-paired* (*slp*) gene (Choe et al. 2006; Choe and Brown 2007).

The PRG ortholog *sloppy-paired* (*slp*) (alternative name *FoxG*) encodes a forkhead-box containing transcription factor, that acts as a secondary PRG in *Drosophila*, where it exists in the form of two paralogs (*slp1* and *slp2*) (Häcker et al. 1992; Grossniklaus et al. 1992). Both paralogs function directly on the SPGs and also act as SPGs. In this function, they are involved in the maintenance of parasegmental boundaries and segment polarity (Cadigan et al. 1994a). In accordance with this dual role, *slp* paralogs are first expressed in the form of seven transverse segmental stripes (PRG pattern), and later in the form of 14 stripes (SPG pattern) (Grossniklaus et al. 1992).

Similar to the two *Drosophila* paralogs, the single described *Tribolium slp* gene appears to function downstream of a regime of primary PRGs (Choe et al. 2006), and is involved in the regulation of SPGs, and thus in segmentation, albeit in a slightly different way than in *Drosophila* (Choe and Brown 2007, 2009).

In this paper, I present the discovery of a second, hitherto unrecognized, paralog of *sloppy-paired* in *Tribolium*. Its embryonic expression pattern suggests a potential role in segmentation, either downstream and/or in parallel with the other *Tribolium* PRG orthologs, including the earlier-described *Tribolium slp* gene (Choe et al. 2006). The presence of a second *slp* gene, designated as *slp2*, may have implications for the interpretation of earlier research conducted on PRG patterning and the gene regulatory network that governs segmentation in *Tribolium*.

## Methods

### *Tribolium* husbandry and preparing embryos

The used specimens of *Tribolium castaneum* stem from the culture in Göttingen/Germany. A colony of this strain was established in Uppsala, following the suggestions made in “The Beetle book” (link: http://wwwuser.gwdg.de/~gbucher1/tribolium-castaneum-beetle-book1.pdf). Embryos were collected and prepared for subsequent in situ hybridization experiments as per Schinko et al. (2009).

### Extraction of total RNA, cDNA synthesis, gene cloning and whole mount in situ hybridization

Total RNA was isolated from complete embryos of mixed developmental stages using TRIZOL (Invitrogen). Total RNA was reverse transcribed into cDNA using the SuperScript First Strand kit (Invitrogen). Gene fragments were isolated by RT-PCR with gene-specific primers. Primer sequences are *slp*_forward: GGTGAAAAGGGAAGAAACGA, *slp*_backward: AGATCACCGTCACTGGTTTA, *slp2*_forward: GGCAGAGAGCAAAGAAACCG and *slp2*_backward: ACAGTGACAGGTTTGAGGAG). Gene fragments were ligated into the PCRII vector (Invitrogen) and sequenced on an ABI3730XL automatic sequencer (Macrogen, Seoul, South Korea). Unique gene identifiers are listed in Supplementary File 1. Monochromatic in situ hybridizations were performed using the standard protocol provided in Janssen et al. (2018). Dichromatic in situ was performed as described in Janssen et al. (2008). Monochromatic in situs were performed with digoxigenin (DIG)-labelled probes; for the second staining in dichromatic in situs, a fluorescein-labelled second probe was used. First, the digoxigenin-labelled probes were detected with BMPurple (Roche), giving rise to a blue signal. After that, alkaline phosphatase on the DIG-labelled antibodies was inactivated with 0.1 M glycine, pH = 2.0 (2-min incubation at room temperature), and the second probe was detected with SIGMAFAST Fast Red TR/Naphtol AS-Mx (Sigma), giving rise to a red signal.

### Identification of potential paralogs of *sloppy-paired* and phylogenetic analysis

Paralogs of *Tribolium sloppy-paired* (*slp*) were identified performing reciprocal BLAST searches (BlastX and BlastP) against the published genomic sequences of the beetle *Tribolium* using the *Drosophila* paralogs *slp1* and *slp2*, and the previously identified *Tribolium slp* gene (Choe et al. 2006) as baits.

Amino acid sequences of the forkhead domains were aligned using T-Coffee (Notredame et al. 2000) using default parameters in MacVector v12.6.0 (MacVector, Inc., Cary, NC). A Bayesian phylogenetic analysis was performed using MrBayes (Huelsenbeck and Ronquist 2001) with a fixed WAG amino acid substitution model with gamma-distributed rate variation across sites (with four rate categories), unconstrained exponential prior probability distribution on branch lengths, and exponential prior for the gamma shape parameters for among-site rate variation. Tree topology was calculated applying 200,000 cycles for the Metropolis-Coupled Markov Chain Monte Carlo (MCMCMC) analysis (four chains; chain-heating temperature of 0.2). Markov chains were sampled every 200 cycles. Default settings were used, defining 25% of the samples as burn-in information. Clade support was calculated with posterior probabilities in MrBayes. Unique identifiers of all used sequences are summarized in Supplementary File 1.

### Data documentation

Pictures of in situ–stained embryos were taken with a Leica DC490 digital camera mounted onto a MZ-FLIII Leica dissection stereo-loupe. Linear adjustments were made on contrast and brightness using the image-processing software Adobe Photoshop CC 2018 for Apple Macintosh (Adobe Systems Inc.)

## Results

### Identification of *Tribolium sloppy-paired* genes

The performed reciprocal BLAST search identified two genes with high sequence similarity to both *Drosophila melanogaster sloppy-paired* genes (*slp1* and *slp2*) (Häcker et al. 1992; Grossniklaus et al. 1992), the single previously described *Tribolium sloppy-paired* gene (*slp*) (Choe et al. 2006), and confirmed *sloppy-paired* orthologs from other arthropods (e.g. Damen et al. 2005, Liu and Patel 2010, Janssen et al. 2011, Green and Akam 2013, Auman and Chipman 2018) and an onychophoran (Janssen and Budd 2013).

**Fig. 1:**
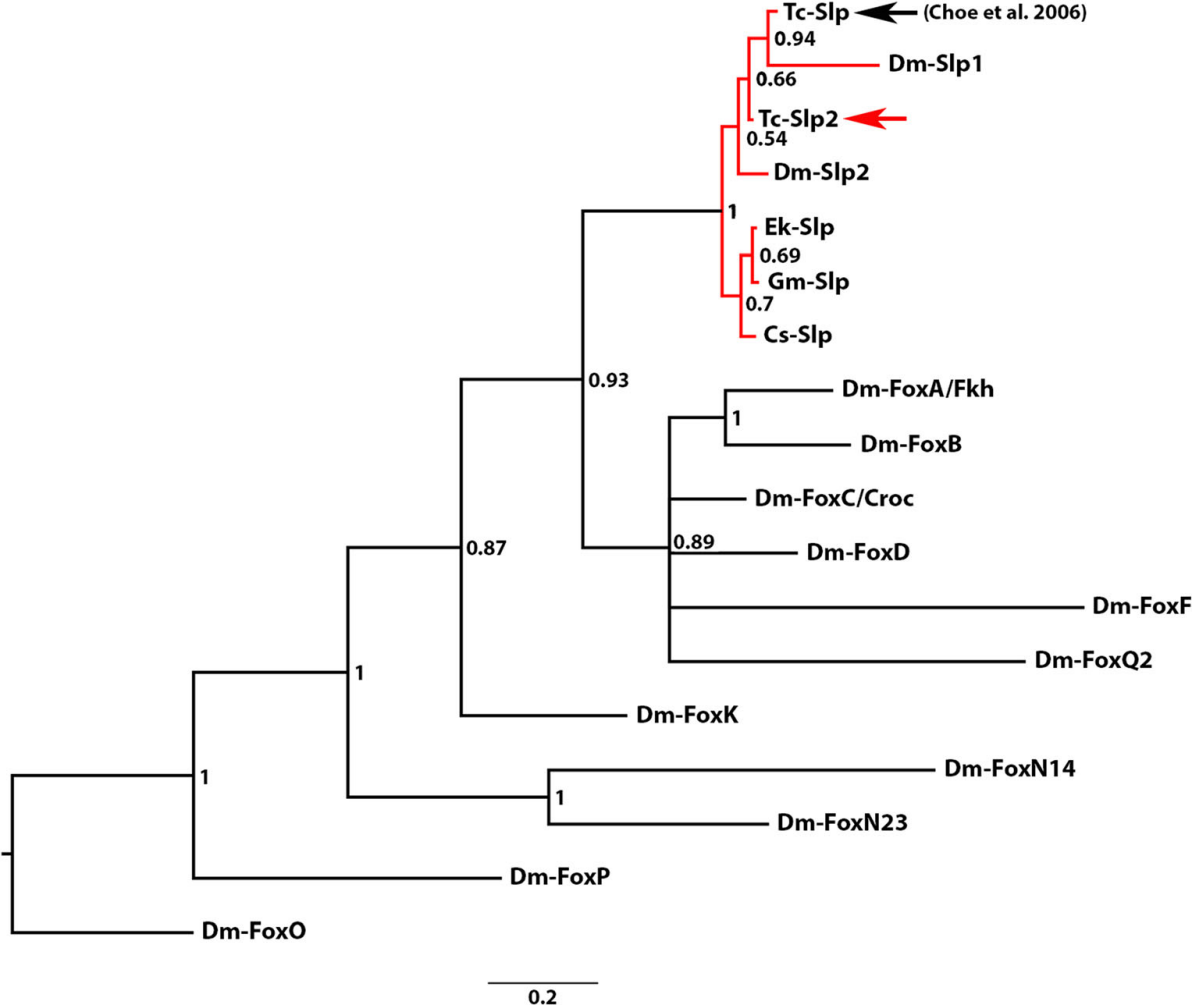
Phylogenetic analysis. Sloppy-paired genes, including the newly identified Slp2, form a monophyletic group with high support (red lines), that is separated from the other *Drosophila* Fox genes. The derived sequence of *Drosophila* FoxO serves as an outgroup. Positions of the two paralogs of *Tribolium sloppy-paired* sequences are marked with arrows. The red arrow points to the newly-discovered Slp2. Species abbreviations: Cs, *Cupiennius salei* (Chelicerata: Aranea); Dm, *Drosophila melanogaster* (Hexapoda: Diptera); Ek, *Euperipatoides kanangrensis* (Onychophora); Gm, *Glomeris marginata* (Myriapoda: Diplopoda), Tc, *Tribolium castaneum* (Hexapoda: Coleoptera)

Phylogenetic analysis shows that these two *Tribolium* genes cluster with panarthropod sloppy-paired orthologs and form a well-supported monophyletic group (Fig. 1). *Tribolium* Slp clusters with high confidence with *Drosophila* Sloppy-paired1 (Slp1), and it seems clear that those two form an orthology-pair. The newly identified *Tribolium* Slp2 and *Drosophila* Slp2 do not form a sister-gene relationship in the phylogenetic analysis. The posterior probability values, however, that separate *Tribolium* and *Drosophila* Slp2 from each another are very low. Therefore, it is possible that the two designated slp2 genes are in fact the result of a duplication at the lineage leading to *Tribolium* + *Drosophila*, and thus could represent paralogs. Note that the distribution of Slp proteins does not represent our current understanding of arthropod relationships.

### Expression of the previously described *Tribolium sloppy-paired (slp)* in comparison with the newly identified second paralog, *slp2*

**Fig. 2:**
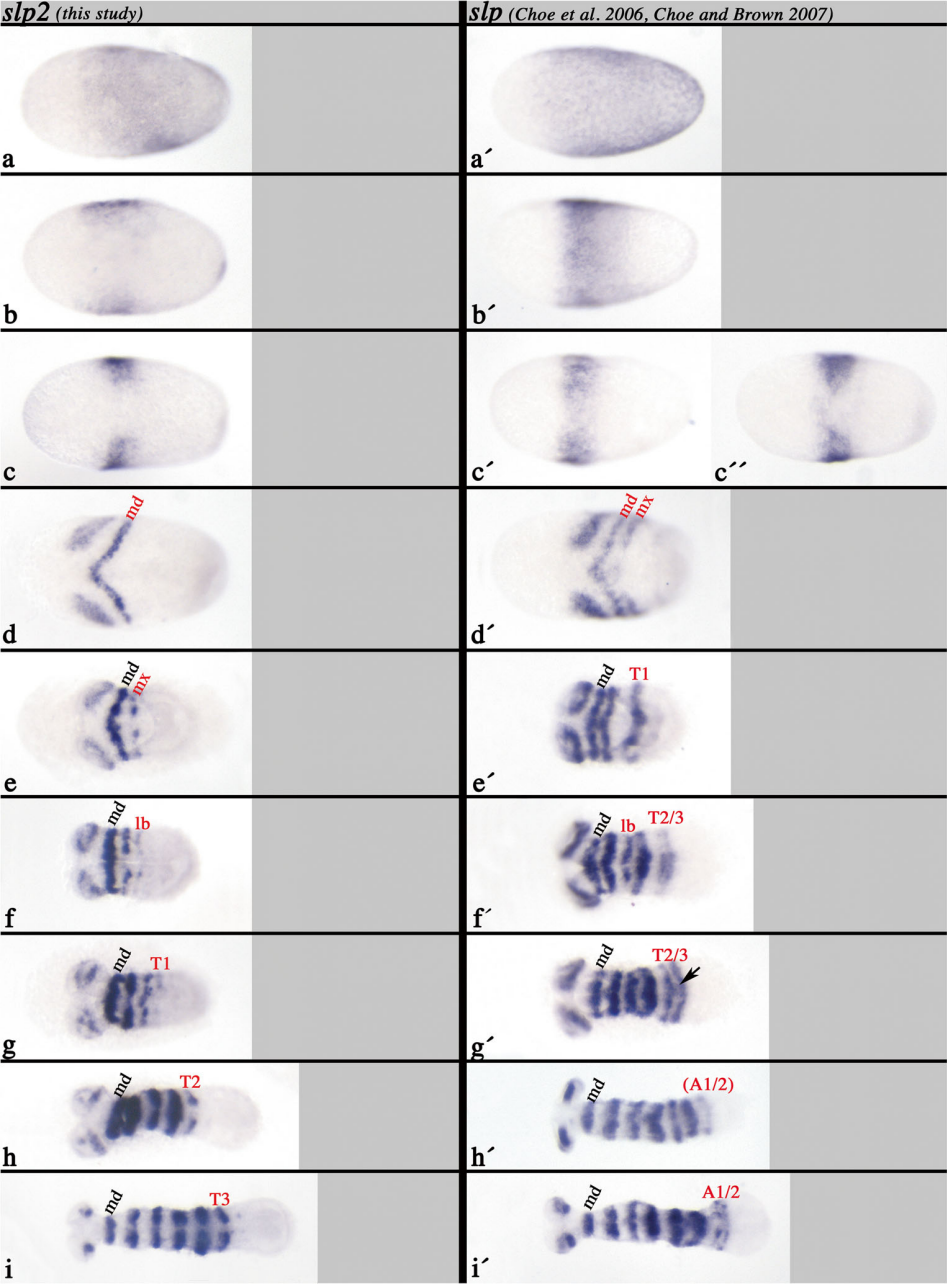

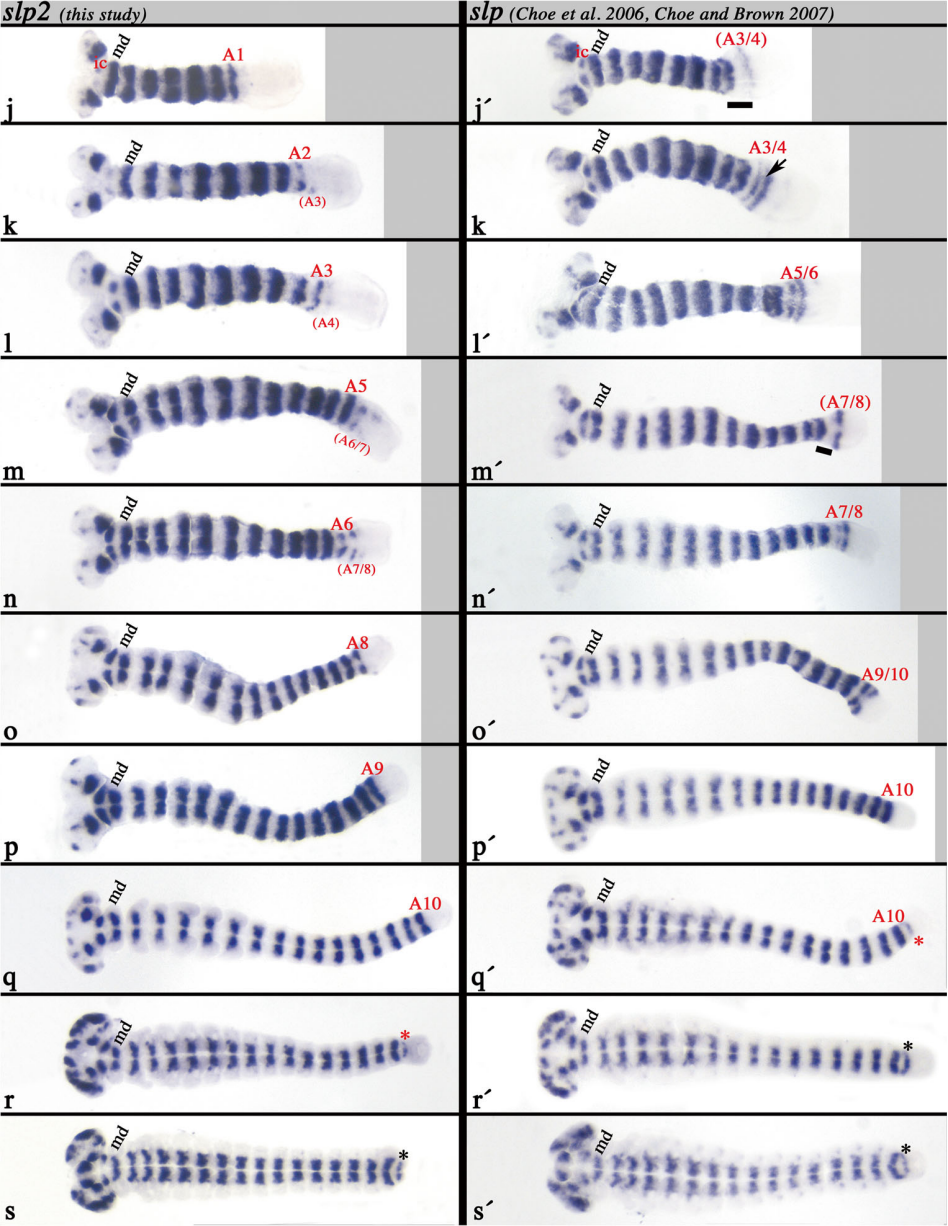
**a** Expression of *slp* (Choe et al. 2006) and the newly identified *slp2*—EARLY STAGES. In all panels, anterior is to the left, ventral views. **a**–**i** Expression of *slp2*. **a´**–**i´** expression of *slp* in comparable stages (as **a**–**i**). Newly formed segments (expression) are indicated in red letters. **a/a´** Early Blastoderm stage. Expression in the posterior 3/5 of the embryo. **b** Later blastoderm stage. Expression retracts from the posterior of the embryo. **c/c´/c´´** Late blastoderm stage. *slp2* is expressed in two domains separated by a ventral gap. *slp* is first expressed in a gap-gene like domain, that later splits ventrally (**c´´**). **d/d´** Gastrulation stage. The gap-gene like domain is split into expression in the future antennal and ocular region. The mandibulary stripe (md) of *slp2* has formed; md and maxillary (mx) stripes of *slp* have formed. Note the simultaneous appearance of two stripes of *slp*. **e/e´***slp2* appears in the mx. *slp* appears in the first thoracic segment (T1). **f/f´***slp2* appears in the labial segment (lb). *slp* appears in lb., and in a broad domain representing T2 and T3. **g/g´***slp* appears in T1. *slp2* expression in T2 and T3 splits into two separate stripes. The arrow points to remnants of expression corresponding to the earlier seen broad domain. **h/h´***slp2* appears in T2. *slp* appears in a broad domain representing the primordia of the first two abdominal segments (A1 and A2). **i/i´***slp2* appears in T3. *slp* expression in A1 and A2 forms separate stripes, **b** Expression of *slp* (Choe et al. 2006) and the newly identified *slp2*—LATER STAGES. In all panels, anterior is to the left, ventral views. **j**–**s** Expression of *slp2*. **j´**–**s´** Expression of *slp* in comparable stages (as **j**–**s**). Newly formed segments (expression) are indicated in red letters. **j/j´***slp2* appears (weakly) in the intercalary segment (ic) and in A1. *slp* appears in ic and the primordia of A3 and A4. Note the space between this new stripe and the last-formed stripe in A4 (black bar). **k/k´***slp2* appears in A2 (and weakly in an anterior to posterior order) in A3. *slp* expression in A3 and A4 forms separate stripes. The arrow points to remnants of expression corresponding to the earlier seen broad domain. **l/l´***slp2* appeared in A3 and (weakly) appears in A4. *slp* is expressed as two stripes representing A5 and A6. **m/m´***slp2* is in A5 and (weakly) appears in A6 and (very weakly) in A7. *slp* appears in the primordium of A8. Note the space between this new stripe and the last-formed stripe in A6 (black bar). **n/n´***slp2* weakly appears in A8. *slp* appears in the primordium of A7 between the stripes in A6 and the primordium of A8. **o/o´***slp2* appeared in A8. *slp* appeared in A9 and A10. **p/p´***slp2* appeared in A9. *slp* is in A10. **q/q´***slp2* appeared in A10. *slp* appears in a domain posterior to A10 (asterisk). **r/r´***slp2* appears in a domain posterior to A10 (asterisk). **s/s´***slp2* and *slp* are expressed in identical segmental patterns

The *Tribolium slp* gene has been identified in Choe et al. (2006), and its expression has been described in some detail in Choe and Brown (2007). Here, I present a more detailed description of *slp* in a series of consecutive developmental stages, revealing some additional aspects of its expression, and at the same time compare its expression with that of *slp2* (Fig. 2).

At the blastoderm stage, both paralogs of slp are expressed in identical patterns in the complete embryo, except for its anterior cap that will later give rise to the serosa (Fig. 2a/a´). In a slightly later blastoderm stage, expression of both paralogs disappears from the posterior of the embryo resulting in a broad central domain of expression (Fig. 2b/b´). In contrast to *slp* (Fig. 2b´), expression of *slp2* is weaker ventrally and finally fully disappears, resulting in two separate domains of expression in the following stage (Fig. 2c). The same ventral disappearance of expression happens with *slp*, but at a slightly later stage (Fig. 2c´´). This is evident from the presence of an intermediate stage in which expression is visible in the form of a solid band (i.e. prior to ventral disappearance) (cf. Fig. 2c´/c´´). With the beginning of gastrulation (Fig. 2d/d´), two stripes of *slp* appear simultaneously and posterior to the anterior expression domain which is located in the head and likely contributes to the anlagen of the antennae and the eyes (see later developmental stages). The two new stripes correspond to the mandibular (md) and maxillary (mx) segment (Fig. 2d´). At the same time, only one stripe of *slp2* appears in the anlage of the mandibles (md) (Fig. 2d). The next stripe of *slp* expression appears in the first thoracic segment (T1) (Fig. 2e´), while *slp2* now appears in the mx (Fig. 2e). *slp* expression then appears in the labial (lb) segment and, in the form of a single broad domain, in the anlagen of the second and the third thoracic segments (T2 and T3) (Fig. 2f´). *slp2*, however, only appears newly in the lb. (Fig. 2f´). The broad domain of *slp* in T2/T3 then separates into two stripes (Fig. 2g´), while for *slp2*, the T1 stripe forms (Fig. 2g). Note the remnants of earlier expression (the broad domain) of *slp2* between the now-forming two stripes (Fig. 2g´, arrow; also see Fig. 2k´). Then another broad posterior domain of *slp* appears which is corresponding to the first two abdominal segments (A1/A2) (Fig. 2h´). At approximately the same time, expression of *slp2* appears in T2 (Fig. 2h). As the posterior expression of *slp* splits into separate stripes in A1 and A2, the T3 stripe of *slp2* appears (Fig. 2i/i´). At the following stage, two new domains of expression appear for both genes. The first is the “delayed” appearance in the intercalary segment (ic) (Fig. 2j/j´). Additionally, another broad domain of *slp* appears representing the third and fourth abdominal segments (A3/A4) (Fig. 2j´), and, at this time, also the first abdominal stripe of *slp2* appears (Fig. 2j). This pattern of posterior stripe-addition and splitting (for *slp*) is maintained in the following developmental stages: addition of A3/A4 (*slp*) and A2 (A3 weakly) (*slp2*) (Fig. 2k/k´), addition of A5/A6 (*slp*) and A3 (A4 weakly) (*slp2*) (Fig. 2l/l´), addition of A7/A8 (*slp*) and A5 (A6 and A7 weakly) (*slp2*) (Fig. 2m/m´), splitting of A7/A8 (*slp*) and A6 (A7 and A8 weakly) (*slp2*) (Fig. 2n/n´), addition of A9/A10 (*slp*) and A8 (*slp2*) (Fig. 2o/o´), splitting of A9 and A10 (*slp*) and addition of A9 (*slp2*) (Fig. 2p/p´). Then, a single stripe of *slp* forms posterior to the A10-stripe (Fig. 2q´), and the A10 stripe of *slp2* forms (Fig. 2q). As this last added stripe of *slp* becomes stronger, the same stripe of *slp2* forms (Fig. 2r/r´ and s/s´).

Expression of *slp* in the labrum appears at the stage depicted in Fig. 2o´. Expression of *slp2* in the labrum, however, appears slightly later in the embryo shown in Fig. 2q.

In summary, segmental expression of *slp* appears in a double-segmental pattern and involves either the splitting of an initial broad domain into two distinct stripes in adjacent segments, or intercalation of a secondary stripe anterior to the last-formed stripe. Stripes of *slp2*, however, appear in an anterior to posterior order with a single-segment periodicity. The segmental stripes of *slp* appear earlier than those of *slp2* (Fig. 2).

**Fig. 3:**
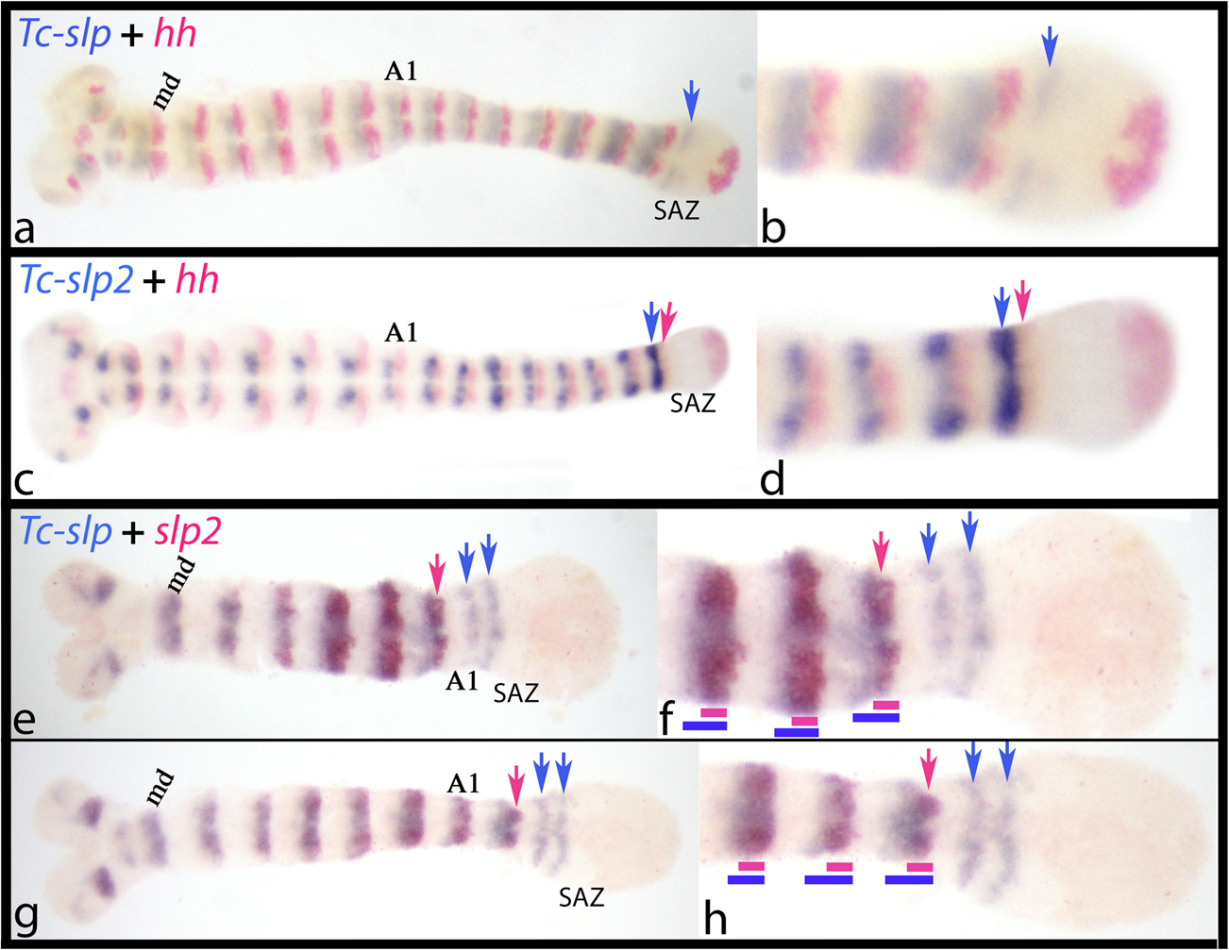
Intra-segmental position of *Tribolium slp* and *slp2*. In all panels, anterior is to the left; ventral views. Panels **a**, **d**, **f** and **h** represent magnifications of the posterior regions of the embryos shown in **a**, **c**, **e** and **g** respectively. Co-expression of *slp* (**a**/**b**, blue signal) and *slp2* (**c**/**d**, blue signal) with the SPG *hedgehog* (*hh*) (red signal) reveals the intra-segmental position of the two *Tribolium slp* genes. Both genes are expressed anterior adjacent to *hh*, and thus are co-expressed with *wingless* (*wg*) in the posterior region of the parasegment. Note that expression of *hh* appears after the expression of *slp* in the anterior of the segment addition zone (SAZ) (blue arrows in panels **a** and **b**), but that *hh* co-appears with the expression of *slp2* (blue arrows in panels **c** and **d**). Co-expression of *slp* (blue signal) with *slp2* (red signal) (**e**–**h**) reveals that *slp* (blue arrows) appears earlier and more posteriorly (in the anterior SAZ) than *slp2* (red arrows) which appears in the last newly forming segment anterior to the SAZ. Note that the expression of *slp* extends somewhat more anteriorly in the segments (blue bars) than *slp2* (red bars). Abbreviations: A1, first abdominal segment; md, mandibular segment

The intra-segmental position of *slp* has been shown to be anteriorly adjacent to the expression of the SPG *engrailed* (*en*) (Choe and Brown 2007). Double expression of *slp* with the downstream target of En, *hedgehog* (*hh*), corroborates this finding (Fig. 3a/b). The intra-segmental position of *slp2* is almost identical with that of *slp* as revealed by co-expression with *hh* (Fig. 3c/d). Double in situs with *slp* and *slp2*, however, reveal that the expression of *slp* extends somewhat more towards anterior than that of *slp2* (Fig. 3e, f, g, h).

With respect to their temporal appearance during segment addition, the appearance of the segmental expression of *slp* predates the onset of *slp2*-expression in stripes (Figs. 2 and 3e, f, g, h).

## Discussion

### The expression profiles of the two *Tribolium* slp genes share common features with their *Drosophila* orthologs

In *Drosophila*, the Sloppy-paired locus contains two copies of the gene, *slp1* and *slp2* (Häcker et al. 1992; Grossniklaus et al. 1992). It is therefore tempting to assume that the presence of two paralogs in *Tribolium* may be correlated with the function(s) of the two *Drosophila* slp genes, i.e. that in both species, *slp1* and *slp2*, respectively, share the same function(s). In *Drosophila*, *slp1* is expressed slightly earlier than its paralog, *slp2* (Grossniklaus et al. 1992; Häcker et al. 1992). Therefore, and although the overall function of the two paralogs is largely redundant, it has been assumed that *slp1* may be responsible for the early function of the Slp locus, i.e. that of a PRG, and that the later expressed *slp2* may be (in concert with *slp1*) involved in the later function, i.e. that of a SPG (Cadigan et al. 1994b).

Interestingly, this situation appears to be conserved in *Tribolium*, where *slp* (the likely ortholog of *Drosophila slp1*) is expressed earlier than its paralog *slp2*. In addition, *slp* is expressed in a PRG-like pattern in the form of initial broad domains that later split and then correspond to expression in two adjacent segments, or indeed by intercalation (discussed below) (Fig. 2). In contrast, *Tribolium slp2* expression is that of a typical SPG (cf. expression of *Tribolium* SPGs; e.g. Farzana and Brown 2008) with a clear single-segment periodicity (Fig. 2). The expression profiles of *Tribolium slp* and *slp2* are thus remarkably similar to those of the two *Drosophila* slp genes, suggesting at least partially conserved regulation and interaction of these genes in the fly and the beetle.

### Pair-rule patterning: Intercalation or splitting?

In *Drosophila*, the secondary PRG-stripes of *slp1* and *slp2* form by intercalation, i.e. de novo formation of stripes in between the first-formed primary PRG-stripes (Grossniklaus et al. 1992). In *Tribolium*, the situation is not that clear. It is obvious that the segmental expression of *slp2* appears with a single-segment periodicity (this study). Posterior stripes of *slp*, however, appear in pairs, or do they not? The mandibular (md) and maxillary (mx) stripes seem to appear as a pair (Fig. 2d) (see also Choe and Brown 2007). In the following labial (lb) segment, however, appearance of *slp* is delayed; this stripe intercalates (like the secondary stripes of *slp1* and *slp2* in *Drosophila*). For the next two pairs of stripes, the situation is unclear. It appears that these stripes form by splitting of an initially broad single domain (Fig. 2f´, g´, h´, i´) (see also Choe and Brown 2007, their Fig. 2g), rather than by intercalation of the secondary (anterior) stripes. A remnant of the initial broad domain (Fig. 2f´) that may document the splitting is present (Fig. 2g´, arrow). Such remnant of expression has been interpreted as evidence of splitting of a PRG’s initial expression in the grasshopper *Schistocerca* (Davis et al. 2001). The formation of stripes in the third and fourth abdominal segments (A3/A4), however, may imply intercalation (note the spacing (black bar) between the last-formed stripe in the embryo shown in Fig. 2j´). However, again, in the following stage, possible remnants of this initial domain are present between the two stripes (Fig. 2k´, arrow). The situation in A7 and A8 is similar, displaying a large distance between a first-formed stripe and the stripe in A6 (Fig. 2m´). It is therefore unclear if the secondary (anterior of each pair) stripes of *slp* appear by intercalation or splitting. Possibly, there are also differences in the regulation of the patterning of anterior abdominal versus posterior abdominal segments as shown for the regulation of the SPG *H15* in this species (Janssen 2014).

Both splitting of initially formed broad double-segment wide stripes and intercalation of secondary stripes as seen in *Drosophila* appear to be a characteristic of PRG expression in general, as it is seen in a wide range of drosophilids, non-drosophilid insects with a long-germ developmental mode and in short-germ arthropods such as *Tribolium*, myriapods and even a mite (e.g. Binner and Sander 1997; Dearden et al. 2002; Chipman et al. 2004; Choe and Brown 2007; Wilson et al. 2010; Janssen et al. 2012). If these double-segmental patterns in the various arthropods are conserved, or have evolved independently, again, remains unclear.

### The intra-segmental position of slp genes in arthropods is conserved

In both the beetle and the fly, both slp genes are expressed in stripes anterior adjacent to *engrailed* (*en*) and *hedgehog* (*hh*), and thus, at least partly, co-expressed with *wingless* (*wg*) (Grossniklaus et al. 1992, Choe and Brown 2007, this study). The relative expression of *Drosophila slp1* and *slp2* in comparison with each other has not been investigated in detail, but the two slp genes are described as fully co-expressed (Grossniklaus et al. 1992, Erik Clark (personal communication)). The broader expression of *slp* in *Tribolium* compared with *slp2* thus represents a difference between these two species. Data on slp expression from other insects are scarce. In another beetle, *Dermestes maculatus*, a single *slp* gene has been identified, that is expressed in a comparable pattern with that of the *Tribolium* slp genes, and plays a classic function as PRG (complementary to that of the other secondary PRG, *paired* (*prd*)) (Xiang et al. 2017). Its intra-segmental position is not clear. In the true bug *Oncopeltus fasciatus*, the single identified *slp* gene is expressed as stripes with a single-segment periodicity in the anterior SAZ and newly forming segments, is under control of the bona fide gap gene *giant* (Liu and Patel 2010) and appears to have a conserved function as segment-polarity gene (Auman and Chipman 2018; Reding et al. 2019). Data on crustacean *slp* expression are not available. In the myriapods *Glomeris marginata* and *Strigamia maritima*, *slp* is expressed with a single-segment periodicity in posteriorly added segments and anterior to *en* (Janssen et al. 2011; Green and Akam 2013). The intra-segmental position is thus conserved between insects and myriapods. Finally, in the spider *Cupiennius salei*, *slp* is expressed in stripes in newly forming segments, but its intra-segmental position is not clear (Damen et al. 2005).

### Possible implications for earlier studies

The finding that *Tribolium*, like *Drosophila*, possesses two slp genes with shared and comparable (to *Drosophila*) expression profiles could explain some of the differences in the regulation and function of *Tribolium slp* reported in earlier studies, and suggests the need for a re-investigation of the function(s) of *slp* and *slp2* in *Tribolium* (Choe and Brown 2007).

In *Drosophila slp* null mutants, expression of *wg* disappears from alternating segments, while in the same segments, expression of *en* extends towards anterior into the natural domain of *wg* (summarized in Choe and Brown 2007). In *Tribolium*, however, downregulation of *slp* leads inter alia to defects in all anterior segments, but in posterior segments, alternating segments are affected (Choe and Brown 2007). Interestingly, however, the register in which *slp* affects segmentation is shifted (the alternating pattern is reversed) in these two species (Choe and Brown 2007). Another difference is that in *Drosophila* and the true bug *Oncopeltus*, but not in *Tribolium*, *en* expands towards anterior in the absence/downregulation of Slp function (Choe and Brown 2007; Auman and Chipman 2018; Reding et al. 2019). While the former cannot easily be explained by the presence of a second paralog in *Tribolium* (i.e. *slp2*), it could be that *en* remains restrained to its natural domain in the absence of *slp* in *Tribolium* because *slp2* acts as a potential repressor of *en*-expression. Alternatively, the remaining low activity of *slp* after RNAi may be responsible for this difference.

## Electronic supplementary material

Supplementary File1 Unique sequence identifiers (DOCX 38 kb)

Supplementary File2 Alignment (MSAP 22 kb)
