# Supplementary material for: The embryonic expression pattern of a second, hitherto unrecognized, paralog of the pair-rule gene sloppy-paired in the beetle Tribolium castaneum
Source: Dev Genes Evol. 2020 May 20;230(3):247–56. doi: 10.1007/s00427-020-00660-x (PMC7260273; doi:10.1007/s00427-020-00660-x)
Supplement: Supplementary file 1 — Unique sequence identifiers (DOCX 38 kb) [file 427_2020_660_MOESM1_ESM.docx]

Gene identifiers

Tc_slp NP_001071091

Tc_slp2 XP_973691

Ek_slp CDF52139

Gm_slp CBX36143

Cs_slp CAI91293

Dm_slp1 NP_476730

Dm_slp2 NP_476834

Dm_FoxA XP_016942864

Dm_FoxB NP_001287516

Dm_FoxC NP_524202

Dm_FoxF NP_523814

Dm_FoxK NP_001261702

Dm_FoxN14 NP_524302

Dm_FoxN23 CAB64654

Dm_FoxO NP_001262557

Dm_FoxP AEQ25432

Dm_FoxQ2 NP_651951
